# Frequency set selection for multi-frequency steady-state visual evoked potential-based brain-computer interfaces

**DOI:** 10.3389/fnins.2022.1057010

**Published:** 2022-12-21

**Authors:** Jing Mu, David B. Grayden, Ying Tan, Denny Oetomo

**Affiliations:** ^1^Department of Mechanical Engineering, The University of Melbourne, Parkville, VIC, Australia; ^2^Department of Biomedical Engineering, The University of Melbourne, Parkville, VIC, Australia; ^3^Graeme Clark Institute for Biomedical Engineering, The University of Melbourne, Parkville, VIC, Australia

**Keywords:** brain-computer interface (BCI), brain-machine interface (BMI), electroencephalography (EEG), dual-frequency, multi-frequency, optimization, steady-state visual evoked potential (SSVEP)

## Abstract

**Objective:**

Multi-frequency steady-state visual evoked potential (SSVEP) stimulation and decoding methods enable the representation of a large number of visual targets in brain-computer interfaces (BCIs). However, unlike traditional single-frequency SSVEP, multi-frequency SSVEP is not yet widely used. One of the key reasons is that the redundancy in the input options requires an additional selection process to define an effective set of frequencies for the interface. This study investigates systematic frequency set selection methods.

**Methods:**

An optimization strategy based on the analysis of the frequency components in the resulting multi-frequency SSVEP is proposed, investigated and compared to existing methods, which are constructed based on the analysis of the stimulation (input) signals. We hypothesized that minimizing the occurrence of common sums in the multi-frequency SSVEP improves the performance of the interface, and that selection by pairs further increases the accuracy compared to selection by frequencies. An experiment with 12 participants was conducted to validate the hypotheses.

**Results:**

Our results demonstrated a statistically significant improvement in decoding accuracy with the proposed optimization strategy based on multi-frequency SSVEP features compared to conventional techniques. Both hypotheses were validated by the experiments.

**Conclusion:**

Performing selection by pairs and minimizing the number of common sums in selection by pairs are effective ways to select suitable frequency sets that improve multi-frequency SSVEP-based BCI accuracies.

**Significance:**

This study provides guidance on frequency set selection in multi-frequency SSVEP. The proposed method in this study shows significant improvement in BCI performance (decoding accuracy) compared to existing methods in the literature.

## 1. Introduction

A steady-state visual evoked potential (SSVEP) is brain activity that frequency-locks to periodic visual stimulation (Zander et al., 2009). SSVEP is widely used in brain-computer interfaces (BCIs) due to its relatively high accuracy and information transfer rate as well as minimal training required of users compared to other modalities, such as motor imagery (Nicolas-Alonso and Gomez-Gil, 2012).

A standard SSVEP-based BCI includes multiple stimuli in the workspace that each flashes at a different frequency while electroencephalography (EEG) is measured primarily from the occipital lobe. The measured EEG reflects the frequency of the stimulus the user is focusing upon visually, as well as the harmonics of that frequency. The presence of the harmonics provides more reference points in the decoding process, but also presents additional complexities and challenges in the design of SSVEP-based BCIs. For example, if a frequency and its harmonic are both used in the same BCI for two different stimuli, there will be common frequencies in the recorded EEG from the two stimuli, which may confound the decoding algorithm. Therefore, in the literature, some studies intentionally avoided the use of frequencies with common harmonics in the stimulation (Volosyak et al., 2009; Chen et al., 2015). This harmonic issue, together with the limited responsive frequency range of the human brain to periodic visual stimulation (Regan, 1989), constrains the number of unique frequencies that can be used in standard SSVEP-based BCIs; i.e., low signal-to-noise ratio EEG recordings and small frequency separation impairs decoding performance. Therefore, it is challenging to use standard SSVEP-based BCIs in scenarios that require large numbers of unique frequencies to label all the targets.

To address this problem, multi-frequency stimulation methods utilizing more than one frequency in each stimulus have been introduced, with two frequencies (dual-frequency) being the most widely used modality (Shyu et al., 2010; Zhang et al., 2012; Chen et al., 2013; Hwang et al., 2013; Kimura et al., 2013; Chang et al., 2014; Mu et al., 2021a). However, these studies focused primarily on introducing multi-frequency stimulation methods, and did not explore frequency selection methods. The use of multiple frequencies on each stimulus or targets can exponentially increase the number of targets that can be represented in the work space as the number of frequencies used to label each target increases. Multi-frequency stimulation generates a complex periodic stimulation signal that triggers a more complex SSVEP response. In Mu et al. (2021a), it was demonstrated that multi-frequency SSVEP response contained not only the input frequencies and their harmonics, but also the integer linear combinations of the input frequencies with low order interactions more likely to be observed in the recorded SSVEP. Note that the order of interaction was defined as the sum of absolute values of the coefficients in the linear combination (Mu et al., 2021a). A dedicated decoding algorithm, multi-frequency canonical correlation analysis (MFCCA), was also introduced to decode complex multi-frequency SSVEP, leveraging the linear combinations produced by the frequency interactions (Mu et al., 2021b). A 20% increase in accuracy was observed when linear combinations of frequencies were utilized to capture the interactions between the input frequencies in the decoding algorithm; however, the study did not investigate whether this information could be used in frequency selection to further improve the performance of the multi-frequency SSVEP-based BCI.

While multi-frequency SSVEP can provide a large number of inputs for the interface, there is a need to select the most effective set of input frequencies to construct a high-performing BCI. In traditional single-frequency SSVEP, frequency selection is usually done following very simple rules: avoid harmonics in the same set (Volosyak et al., 2009), as mentioned above, and avoid small frequency intervals. In studies with a relatively large number of targets (40 targets) by Chen et al. (2015), Wang et al. (2016), and Liu et al. (2020), stimulation frequencies were equidistantly selected in a range (8–15.8 Hz with 0.2 Hz intervals) that avoided the existence of harmonics in the same range. Another common way to select frequencies in constructing an SSVEP-based BCI is based on the refresh rate of the screen (refresh rate divided by integer numbers; Bakardjian et al., 2010; Zhu et al., 2010; Hwang et al., 2013). In dual-frequency SSVEP, this problem was explored by minimizing the maximum input (stimulation) signal correlation (Liang et al., 2020). Although this optimization method demonstrated its advantage in improving the accuracy of the interface, only the optimization of input signals was investigated. Since multi-frequency SSVEP response shows more complex frequency interactions between the multiple frequencies used in the stimulation, optimization that takes into account such frequency interactions at the output signals may outperform techniques that only take into account the stimulation signals.

This work built upon Liang et al. (2020) and investigated whether optimizing on a known multi-frequency SSVEP feature would result in different performance in the multi-frequency SSVEP-based BCI. In this paper, the dual-frequency SSVEP is considered as a special case of multi-frequency SSVEP. Under such a setting, an optimization strategy based on not only the input frequencies, but also their harmonics and the integer linear combinations of the input frequencies, is proposed and tested. The number of frequencies used in constructing the targets, i.e., whether to select frequencies to make all the pairs or to select pairs directly from the range without any constraint, is also considered along with the output-based optimization. This work contributes toward the framework of increasing the number of commands in “BCI as a processor,” where command processing capacity is a key (Xu et al., 2021).

## 2. Materials and methods

### 2.1. Definitions

To avoid confusion, some terms used in this paper are defined below.

#### Selection by frequencies

A frequency selection approach where the minimum number of frequencies in the given range are selected to construct the required number of targets.

#### Selection by pairs

Select the required number of pairs directly from the given frequency range (from all the possible pairs made by the frequencies within the range). Selection by pairs provides more freedom in frequency selection as opposed to selection by frequencies. Selection by pairs ignores additional constraints the system may have, for example, limited number of frequencies that can be produced by the hardware, in which case selection by frequencies would be preferred.

#### Frequency pair

In a dual-frequency application, the frequency pair refers to the two stimulation frequencies used to represent one target.

#### Frequency set

The set of all frequency pairs used in the interface.

#### Common sums

The common frequencies resulted from the integer linear combinations of different pairs of frequencies, where at least one of the coefficients is not zero. The number of common sums refers to the counted number of common sums between the frequency pairs in the frequency set [bounded by order, where order is the sum of absolute values of the coefficients in the linear combination (Mu et al., 2021b)].

#### Optimization strategy

In this paper, optimization strategy refers to the optimization problem formulations, which includes the cost function and the parameters to tune.

The following example is provided to help illustrate use of the terms. Given a 6-target setup and five frequencies to select from, selection by frequencies selects four frequencies out of the five because it needs at least four frequencies to make six targets with dual-frequency stimulation ($C_{2}^{4}=6$). For example, the five frequencies are 5, 6, 7, 8, and 9 Hz. The frequencies 5, 6, 7, and 9 Hz are selected from selection by frequencies, so the resulting frequency set will be {(5, 6), (5, 7), (5, 9), (6, 7), (6, 9), (7, 9)}, where each parenthesis includes one frequency pair; for example, (5, 6). On the other hand, selection by pairs selects 6 pairs from the $C_{2}^{5}=10$ total pairs of frequencies available. An example of the selected frequency set from selection by pairs could be {(5, 6), (5, 8), (5, 9), (6, 7), (6, 8), (8, 9)}.

Common sums can be found among the frequency pairs, for example between {(5, 7), (7, 9)}. Table 1 lists all the linear combinations (up to order 2) from the 2 frequency pairs. We can see from the table that there are three frequencies that are common to the two pairs (the common sums): 7, 14, and 2. Therefore, the number of common sums in the frequency set {(5, 7), (7, 9)} is 3. Note: the term “order” refers to the sum of the absolute values of the coefficients in the linear combination.

**Table 1:** Integer linear combinations up to order 2 in frequency set {(5, 7), (7, 9)}.

**Operation**		***f*_1_**	***f*_2_**	**2 × *f*_1_**	**2 × *f*_1_**	***f*_1_+*f*_2_**	**|*f*_1_−*f*_2_|**
Frequency pairs	(5, 7)	5	7	10	14	12	2
(*f*_1_, *f*_2_)	(7, 9)	7	9	14	18	16	2

### 2.2. Hypotheses on frequency set selections

In multi-frequency SSVEP, the resulting brain response shows not only the input frequencies and their harmonics, but also the interactions between the input frequencies, in the form of the integer linear combinations of the input frequencies (Mu et al., 2021a). These peak occurrences in the SSVEP response increase the chances of the common sums as described above. Common sums are significant as they introduce ambiguity as to which frequency pair produces an identified SSVEP peak during decoding. Hence, we expect to see an increase in decoding accuracy when the number of common sums in the multi-frequency SSVEP is reduced. It is also worth noting that, in some SSVEP-based BCI setups, there are additional constraints, such as a limited number of frequencies that can be produced by the hardware. Therefore, we also consider the case where only the minimum number of frequencies needed for constructing all the targets can be selected (selection by frequencies). Selection by frequencies has more constraints compared to selection by pairs, so a less optimal result was expected, whereas the result from selection by pairs is anticipated to be closer to optimal. Therefore, in this work, two hypotheses on frequency set selection in dual-frequency SSVEP are tested:

Hypothesis 1. The performance (accuracy) of the multi-frequency SSVEP-based BCI will be improved when the number of common sums is minimized in the selected frequency set.
Hypothesis 2. In frequency set selection in multi-frequency SSVEP-based BCIs, selection by pairs results in better performance than selection by frequencies.

In order to test the two hypotheses, four cases are considered:

1. Selection by frequencies without minimizing the number of common sums;
2. Selection by frequencies by minimizing the number of common sums;
3. Selection by pairs without minimizing the number of common sums;
4. Selection by pairs by minimizing the number of common sums.

We have added a fifth case to the list — selection by pairs by maximizing the number of common sums — to examine the efficiency of optimizing on the number of common sums.

### 2.3. Frequency set selection methods

In this work, a frequency range of 11–16 Hz and a 15-target arrangement were used. Single-frequency SSVEP was tested as a benchmark.

The test range 11–16 Hz was selected because it is within the most responsive range of SSVEP (5–25 Hz; Regan, 1989), large enough to test a couple of scenarios but keeping the experiment duration manageable and optimization not too computationally heavy.

#### 2.3.1. Single-frequency

The frequencies selected was evenly spaced within the range with a varying 0.3 or 0.4 Hz interval between the frequencies: [11.3, 11.7, 12.0, 12.3, 12.7, 13.0, …, 15.7, 16.0].

#### 2.3.2. Dual-frequency

The entire dual-frequency set selection is based heavily on the two hypotheses, which made the five cases for us to consider. Therefore, we decided upon five methods in frequency set selection. Table 2 illustrates the relationship between these five methods and the two hypotheses. Details about the five methods will be provided later in this section.

**Table 2:** Frequency set selection methods and the hypotheses.

	**Not minimizing the number of common sums**	**Minimize the number of common sums**	**Maximize the number of common sums**
**Selection by frequencies**	Method 1	Method 2	
**Selection by pairs**	Method 5	Method 3	Method 4

The decision tree method was selected for optimization because it produces a similar result to the global search method and is fast to calculate (Liang et al., 2020). In the decision tree method, two matrices are first constructed. A matrix **A** is constructed of size *N*×*N*, where *N* is the total number of pairs available, and the value in each cell is the optimization parameter between the two pairs whose indices are the row and column indices. In our case, this is the number of common sums between the two pairs. The other matrix is **B** that is initialized to be a zero matrix of the same size as **A**. In each optimization iteration, the minimum (or maximum, if maximizing) value in **A** is first identified, then the value in this cell is updated to a large (or small) value to avoid being selected in the following iterations, and the value in the corresponding location in **B** is set to 1 (or any non-zero value). The **B** matrix then goes through a check to see if there exists a reduced **B** matrix, **B′**, of size *N*_*T*_ that has all elements non-zero. If there exists a **B′** matrix that does not contain any zero elements, then the optimization is done and the resultant frequency set is made up of the frequency pairs whose indices are the selected rows and columns in the **B** to **B′** reduction. Otherwise, continue to the next iteration.

The number of common sums is calculated as the number of times the linear combination frequencies are repeated in the frequency set between the pairs up to a given order. In other words, the number of common sums is the number of times a frequency is repeated in the list of integer linear combinations of all the pairs in the frequency set up to a given order, and only the between-pairs repetitions are counted, any repetitions within a pair is ignored.

Since we are using a 15-target setup, six frequencies should be selected when selecting frequencies ($C_{2}^{6}=15$) and 15 pairs of frequencies should be selected when selecting pairs. To ensure there are sufficient numbers of frequencies to select from and not to over-complicate this problem, the frequency candidates were designed to be 0.5 Hz apart within the range 11–16 Hz; i.e., [11.0, 11.5, 12.0, 12.5, 13.0, 13.5, 14.0, 14.5, 15.0, 15.5, 16.0].

The methods used in this study are listed below:

Method 1: Selection by frequencies with evenly spaced frequency interval. Thus no effort was made to minimize the number of common sum.Frequencies are selected from the full range with even intervals (on the six integers).
Method 2: Selection by frequencies with unevenly spaced frequency interval, which was designed to enable the minimization of the number of common sum.Frequencies are selected from the range with minimized number of common sums. The number of common sums is first checked at order 2, the pairs with the smallest number of common sums are then checked at order 3, etc. We bounded this process to check up to order 5.
Method 3: Selection by pairs with decision tree method (Liang et al., 2020) optimizing (minimizing) the number of common sums between the pairs.Frequency pairs are selected from all possible pairs made by the frequency candidates with minimized number of common sums using decision tree. The common sums are calculated up to order 5.
Method 4: Selection by pairs with decision tree method maximizing the number of common sums between the pairs.Frequency pairs are selected from all possible pairs made by the frequency candidates to maximize the occurrences of common sums using decision tree. The common sums are calculated up to order 5.
Method 5: Selection by pairs with decision tree method minimizing the maximum correlation between the input signals constructed with the pairs (Liang et al., 2020). Frequency pairs are selected from all possible pairs made by the frequency candidates with minimized maximum correlations between the input signals using decision tree.

The resulting frequency sets and the their numbers of common sums are listed in Table 3.

**Table 3:** Frequency sets obtained from optimization and their corresponding number of common sums.

**Method**	**Frequencies/frequency pairs**						**Number of common sums**
**Selection by frequencies**							
1	11.0	12.0	13.0	14.0	15.0	16.0	355
2	11.0	12.0	12.5	14.5	15.5	16.0	282
**Selection by pairs**							
3	(11.0, 14.5)	(11.5, 14.0)	(11.5, 15.0)	(12.0, 14.5)	(12.0, 15.5)		285
	(12.5, 15.0)	(12.5, 16.0)	(13.0, 15.5)	(11.5, 15.5)	(12.0, 16.0)	
	(13.5, 16.0)	(11.0, 11.5)	(11.0, 15.5)	(11.5, 16.0)	(15.5, 16.0)	
4	(11.0, 11.5)	(11.0, 12.5)	(11.0, 13.0)	(11.0, 13.5)	(11.5, 13.0)		311
	(12.0, 12.5)	(12.0, 13.0)	(12.5, 15.0)	(13.5, 16.0)	(14.0, 15.5)	
	(14.0, 16.0)	(14.5, 15.0)	(14.5, 16.0)	(15.0, 15.5)	(15.5, 16.0)	
5	(11.5, 13.5)	(11.5, 15.5)	(11.5, 12.0)	(11.5, 14.5)	(13.5, 14.5)		301
	(14.5, 15.0)	(14.5, 16.0)	(15.5, 16.0)	(11.5, 15.0)	(12.5, 14.5)	
	(12.5, 13.5)	(12.5, 16.0)	(13.5, 16.0)	(11.0, 11.5)	(13.5, 14.0)	

### 2.4. Experimental setup

In the experiments, participants sat 70 cm away from a computer screen where the stimuli were shown. Participants were positioned to be centered to the screen and height adjusted to a comfortable level. All experiments were done in a dim, quiet room.

#### 2.4.1. Stimulation methods

**Figure 1:**
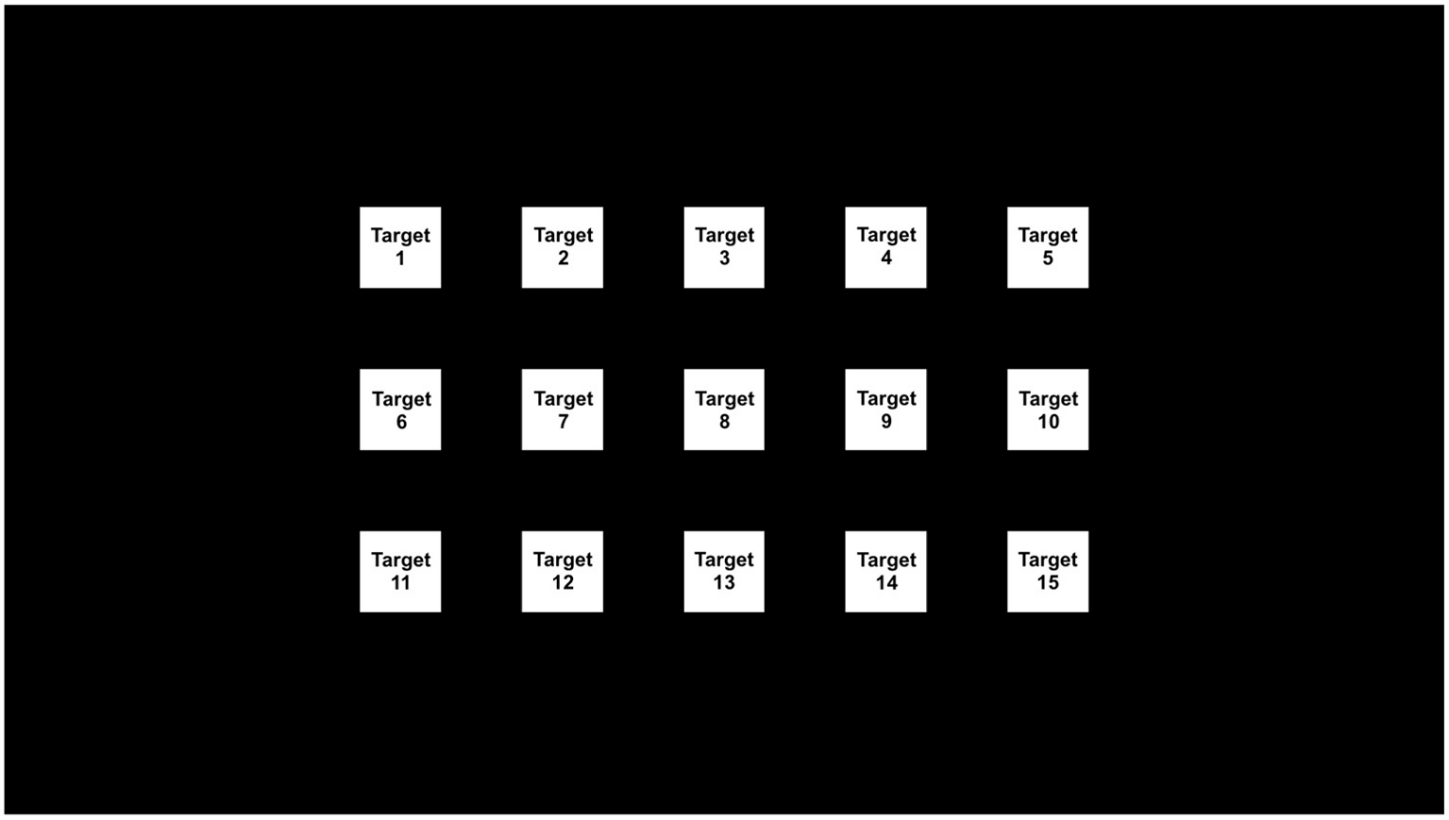
Stimuli layout on the screen.

Visual stimulation was delivered through an Alienware monitor AW2518HF (24.5 inch, 1920 × 1080) running Unity programmed interface at 120 Hz. White color was used for all stimuli. The size of each stimulus was 108 × 108 pixels; the distance between adjacent stimuli was 108 pixels both horizontally and vertically. The 15 targets followed a 3 by 5 layout on the screen as shown in Figure 1.

Single-frequency stimulation was performed by presenting 50% duty cycle square waves on the screen at full brightness. All signals commenced with zero phase shift.

In this work, we have chosen frequency superposition (Mu et al., 2021a) as the multi-frequency stimulation method for its simplicity. Multi-frequency SSVEP stimulated with frequency superposition contains not only the input frequencies and their harmonics, but also the integer linear combinations of the input frequencies, with lower order interactions are more likely to be observed (Mu et al., 2021a,b). Considering the use of input frequencies with narrow frequency gaps (0.5 Hz), ADD logic with equal brightness distribution was selected for superimposing the input frequencies; i.e., in the dual-frequency case, the two input frequencies each correspond to half of the full brightness and the superimposed signal becomes the stimulation signal. Square waves at 50% duty cycle and zero phase shift were used in frequency superposition in this work.

#### 2.4.2. Data acquisition

EEG data was recorded with g.USBamp EEG system and g.SAHARA dry electrodes (g.tec medical engineering GmbH, Austria). The recorded EEG signals were sampled at 512 Hz, with 50 Hz notch and 0.5 − 100 Hz band pass filters on all channels. A 16-channel measurement was taken (P3, Pz, P4, PO3, POz, PO4, O1, Oz, O2, Fz, FCz, FC1, FC2, Cz, C1, and C2); however, only the first nine channels were used for SSVEP processing as these are closest to the visual cortex. Reference and ground electrodes were placed at the left and right mastoids, respectively.

#### 2.4.3. Participants

Fifteen participants participated in the frequency selection experiment; however, three of them did not complete the experiment due to extremely low accuracy experienced in the experiment (on average below three out of 15 trials correct, which makes chance a factor that heavily affected the results). Therefore, data from 12 participants (nine males, three females) aged 22–34 years (28.08 ± 3.70) were included in the analysis.

The experiments were approved by the University of Melbourne Human Research Ethics Committee (Ethics ID: 1851283). Written consent was collected from all participants prior to the experiment.

### 2.5. Experimental protocols

**Figure 2:**
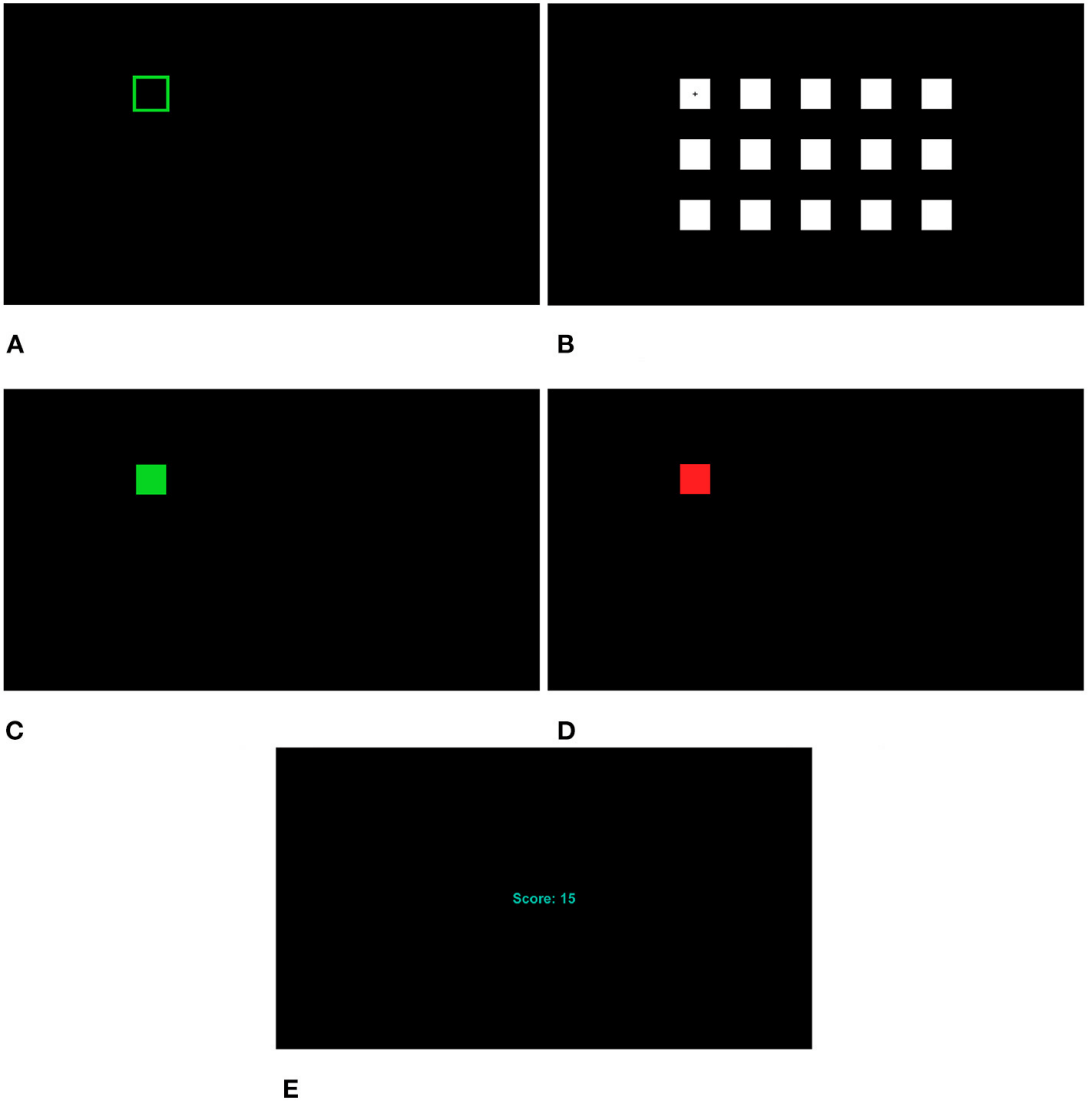
Interface at different stages. **(A)** Cue. **(B)** Fixation cross. All targets are flashing during stimulation. **(C)** Feedback (correct). **(D)** Feedback (incorrect). **(E)** Score.

The experiment contained four sessions with each session having six tests that evaluated the six stimulation frequency setups (five dual-frequency and one single-frequency) once each. Each test had 15 trials (15 targets, one trial per target). Each trial started with a 1 s cue (green outline at intended target, Figure 2A), followed by 5 s stimulation (with a fixation cross at the center of the intended target, Figure 2B, all targets are flashing during this stimulation period), then 1 s feedback (solid green or red square for successful or erroneous identification, respectively, Figures 2C, D), and 1 s rest. A score was shown to the participant after each completed test indicating the number of correct trials for the test (Figure 2E) with 0 indicating none of the 15 trials was identified correctly and 15 indicating all trials were correctly identified. 1 min breaks were provided after each test, 5–10 min breaks were placed between the sessions. The length of breaks were adjusted to the participant's need.

In each test, the participant was asked to go through each of the 15 targets one-by-one following the cue. To simplify the participant's task, in each test, the trial sequence was always from left-to-right, top-to-bottom, going through the targets in target index ascending order (Figure 1). However, the stimulation frequencies or frequency pairs were randomly shuffled among the 15 targets.

**Figure 3:**
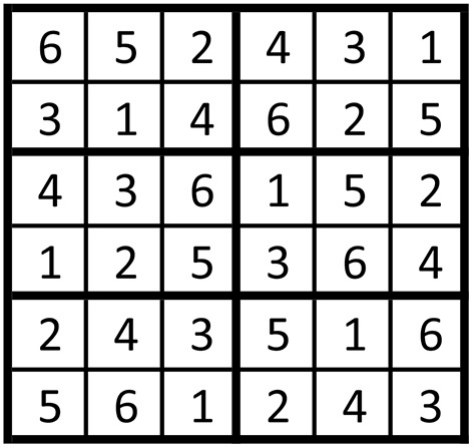
Example of a 6-by-6 Sudoku in brickwall style.

To ensure the experimental results were not affected by user fatigue, Sudokus were used to generate randomized yet balanced test sequences. With six setups to test in this experiment, 6-by-6 Sudokus in brickwall style, as shown in Figure 3, were used. Numbers 1–5 in the Sudoku match to methods 1–5 in dual-frequency selections, and 6 matches to the single-frequency setup. These six different setups will be henceforth referred to as test 1, 2, …, 6.

The experiments were arranged so that each participant used one row in each Sudoku and each session had a different Sudoku. Therefore, with four sessions in the experiment, at least four Sudokus were needed. In order to accommodate 12 participants, eight Sudokus were used in total in this experiment to make 12 rows for the 12 participants.

Participants were trained with up to two sessions with six tests (one test per setup) in each session in a random sequence before the experiment.

### 2.6. Data processing

During the experiments, 5 s of data was used for decoding to produce feedback to the participants. Canonical correlation analysis (CCA; Lin et al., 2006) with number of harmonics set to 3 was used in single-frequency SSVEP decoding. Multi-frequency canonical correlation analysis (MFCCA; Mu et al., 2021b) with order set to 1 was used in dual-frequency SSVEP decoding. The frequency candidate that gave the highest correlation was taken as the decoder output.

Information Transfer Rate (ITR) is often reported in SSVEP experiment results, defined as


(1) $$ITR=\frac{60}{T}\left[\log_{2}N+p\log_{2}p+(1-p)\log_{2}(\frac{1-p}{N-1})\right]\;(bits/min),$$

where *T* is the time window (seconds) for a trial or time needed to produce one result, *N* is the number of targets or possible choices (*N*∈ℤ, *N*≥2), *p* is the mean accuracy (*p*∈(0, 1)); (Wolpaw et al., 2000). In this work, since a consistent setting (15 targets; trials consisted of 1 s cue, 5 s stimulation, 1 s feedback, and 1 s break) was used throughout both experiments, ITR is a static conversion from accuracy and so conveys equivalent information. Therefore, results are mainly shown in terms of accuracy.

## 3. Results

**Figure 4:**
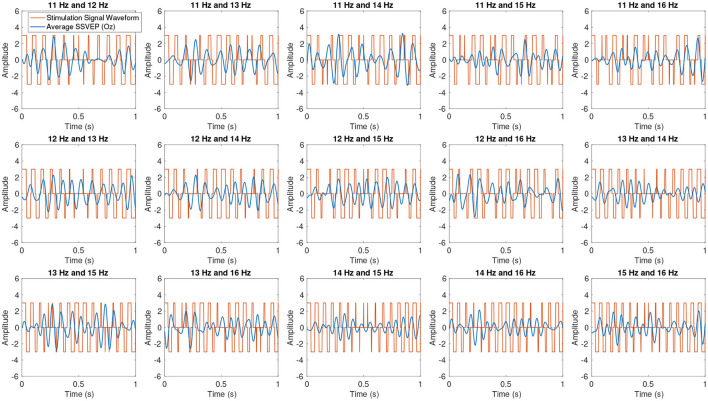
Average SSVEP from Test 1 in time domain. Blue lines plot the average SSVEPs from channel Oz after bandpass filtered between 9 and 18 Hz. Orange lines show the waveforms of the dual-frequency stimulation signals.

**Figure 5:**
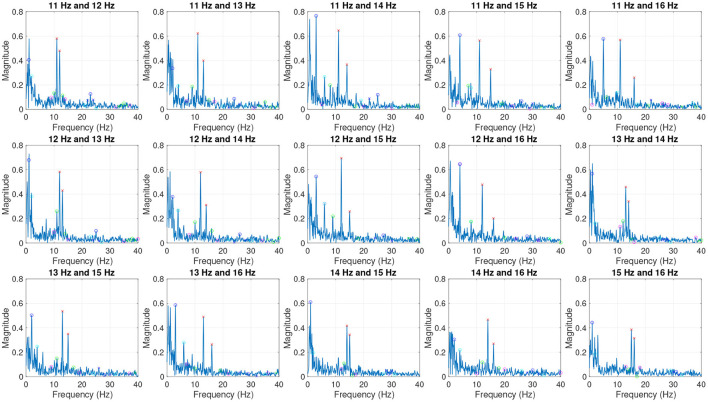
Average SSVEP from Test 1 in frequency domain. Red crosses label the two stimulation frequencies. Crosses and circles indicate harmonics and linear combinations of the two frequencies, respectively. Orders *N*_O_ are shown with different colors: red–*N*_O_ = 1; blue–*N*_O_ = 2; green–*N*_O_ = 3; cyan–*N*_O_ = 4; magenta–*N*_O_ = 5.

The average dual-frequency SSVEPs recorded in test 1 were plotted in both time domain and frequency-domain as shown in Figures 4, 5. The plots shown in the figures are averaged across all participants and all sessions. Figure 4 shows the first second (starting from stimulation onset) of the averaged filtered SSVEP (blue) in comparison to the waveform of the stimulation signal (orange). The SSVEPs recorded from channel Oz were bandpass filtered between 9 and 18 Hz using Matlab function “bandpass” with “ImpulseResponse” set to “auto,” 0.85 “Steepness,” and 60 dB “StopbandAttenuation,” then averaged across all participants and all sessions. Figure 5 plots the averaged SSVEP recorded from channel Oz in frequency domain. The crosses label the two stimulation frequencies and their harmonics, circles label the linear interactions (integer linear combinations) of the two frequencies. The harmonics and linear interactions at different orders *N*_O_ (sum of absolute values of the coefficients in the integer linear combination Mu et al., 2021b) are labeled with different colors as explained in the figure caption.

**Figure 6:**
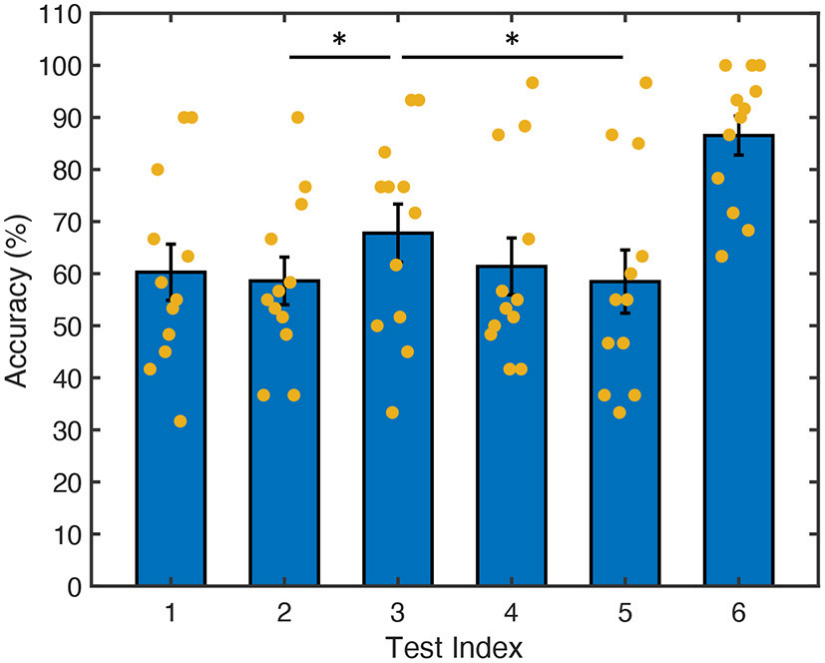
Accuracy from the six tests in frequency set selection experiment. Heights of the bars represent the mean accuracy, error bars show standard error of the average accuracies from 12 participants, and yellow dots label the average accuracy from each participant. * Indicates significant difference (*p* < 0.05, corrected) pair-wise between each two tests with ANOVA. Test 1: dual-frequency method 1; test 2: dual-frequency method 2; test 3: dual-frequency method 3; test 4: dual-frequency method 4; test 5: dual-frequency method 5; test 6: single-frequency.

Figure 6 plots the average accuracies and standard errors for the frequency set selection experiment. The yellow dots in the figure give the average accuracies for each subject. Tests 1–5 are dual-frequency methods with tests 1 and 2 implementing selection by frequencies and tests 3–5 implementing selection by pairs, and test 6 is single-frequency. Figure 6 shows that dual-frequency setups have similar accuracies, except test 3, where the number of common sums is minimized in selection by pairs. All dual-frequency tests (tests 1–5) showed a lower average accuracy compared to the single-frequency test (test 6).

Three-factor analyses of variance (ANOVAs) were performed using data from tests 1–5 with factors subject, test, and session, and the data was fitted using a linear mixed model. The normality of data was confirmed with box plots. Tukey correction was applied to correct for multiple comparisons in the family of five estimates (tests 1–5). Significant differences (adjusted *p*-value *p*_*adj*_ < 0.05) were found between tests 2 and 3 (*p*_*adj*_ = 0.02), and between tests 3 and 5 (*p*_*adj*_ = 0.019), as labeled in the figure with *.

Table 4 lists the ITR from the six tests calculated based on 8s trial duration and 15 targets. Note that the focus of this work is not to increase ITR, but rather to explore and compare different frequency set selection methods in multi-frequency SSVEP.

**Table 4:** Information transfer rate (ITR) from the six tests (mean ± standard deviation).

**Test**	**1**	**2**	**3**	**4**	**5**	**6**
ITR (bits/min)	11.5 ± 6.6	10.7 ± 5.5	14.2 ± 7.0	11.9 ± 7.3	11.1 ± 7.6	22.0 ± 6.3

### 3.1. Effectiveness of minimizing the number of common sums

Our first hypothesis was that minimizing the number of common sums in the frequency set can improve performance. Here, we will check the effectiveness of minimizing the number of common sums in both selection by frequencies and selection by pairs cases.

#### 3.1.1. Selection by frequencies: Even vs. uneven frequency interval

As described in Section 2.3.2, both Method 1 (selection by frequencies with even frequency intervals) and Method 2 (selection by pairs with minimized number of common sums) were on selection by frequencies; Method 1 uses an even frequency interval of 1 Hz and Method 2 selects the same number of frequencies in the range with 0.5 Hz frequency interval, which naturally gives uneven frequency intervals in the selected frequency list. Since Method 1 selects frequencies evenly in the full frequency range, there is no possibility of optimization in Method 1. Method 2 selects frequencies from all candidates using optimization (minimization) on the number of common sums. From Figure 6, we can see that Method 1 did not have a significantly different accuracy to Method 2 (*p*_*adj*_ = 0.98). However, there are actually two factors that contribute to this result: one is the optimization on the number of common sums, the other is the frequency interval in the selected frequencies. To make a comparison between even and uneven frequency selection in the same frequency range, it is unavoidable to have different frequency intervals in the two frequency sets. The uneven frequency interval, while it minimizes the number of common sums, may result in some intervals that are narrower than that employed in the evenly spaced frequency selection method (Method 1). Therefore, the potential advantage of the optimization may be reduced by the smaller frequency interval.

#### 3.1.2. Selection by pairs: Effectiveness of minimizing the number of common sums compared to minimizing maximum correlation

In all selection by pairs methods (Methods 3–5), a consistent 0.5 Hz frequency interval was used. Therefore, the comparison here potentially reflects the effectiveness of minimizing the number of common sums better.

Methods 3 and 5 are based on different optimization strategies: Method 3 optimizes (minimizes) on the number of common sums while Method 5 optimizes (minimizes) on the maximum correlation between input stimulation signals. Hence, a comparison between Methods 3 and 5 demonstrates the effectiveness of minimizing the number of common sums. It can be seen in Figure 6 that the accuracy of Method 3 was significantly (*p*_*adj*_ = 0.019) higher than Method 5 by about 10%. This shows that minimizing the number of common sums is an effective optimization strategy in selecting frequency sets with selection by pairs, at least in dual-frequency SSVEP using frequency superposition with ADD, where the two single-frequency square-wave signals each corresponds to half brightness are superimposed on the target.

While both Methods 3 and 5 use decision trees to perform selection by pairs, the fundamental difference between these two optimization strategies is the parameter that is being optimized. Method 3 optimizes (minimizes) the number of common sums, focusing on the frequency domain characteristics of the input/stimulation signals. Method 5 minimizes the maximum correlation between input (stimulation) signals, focusing on the time domain characterization of the expected SSVEP response elicited by the multi-frequency stimulations. Since the brain is a highly non-linear system, optimizing the system output (EEG signals) might further reduce the confusion in the decoding process, hence the difference.

### 3.2. Selection by frequency vs. selection by pairs with number of common sum minimized frequency selection

The second hypothesis was that selection by pairs results in better performance than selection by frequencies. Here, we compare Methods 2 and 3 as both were optimized (minimized) on the number of common sums and used 0.5 Hz frequency intervals.

The result from Figure 6 shows that Method 3 had a significantly (*p*_*adj*_ = 0.02) higher average accuracy compared to Method 2. This showed that, when we have the freedom to select pairs freely in the whole range of frequencies, we could achieve higher accuracy in the interface compared to when we were constrained on the number of frequencies that could be selected. This difference might be amplified when there are larger numbers of frequencies in the candidate set, more frequencies superimposed on the same target, and/or numbers of targets (*N*_*T*_) increase, because the number of frequency pairs we can select from (*N*_*F*_) and the number of possible frequency sets becomes larger as shown in Equations 2, 3.


(2) $$\begin{array}{c} N_{F,\text{pair}}=C_{N}^{N_{F}}, \end{array}$$

(3) $$\begin{array}{c} N_{F,\text{set}}=C_{N_{T}}^{N_{F,\text{pair}}}, \end{array}$$

where *N*_*F*, pair_ is the total number of possible frequency pairs, *N*_*F*, set_ is the total number of possible frequency sets, *N* is the number of frequencies superimposed on each target.

### 3.3. Efficiency of number of common sum minimization

Methods 3 and 4 are explicitly designed to test the efficiency of number of common sum minimization; Method 3 minimizes the number of common sum and Method 4 maximizes the number of common sum. The result from Figure 6 showed an insignificant difference between the average accuracy of the two tests after adjustment for multiple comparisons (*p*_*adj*_ = 0.21), which is unexpected. This will be discussed in Discussion section.

## 4. Discussion

### 4.1. Efficiency of number of common sum minimization

It is interesting to see that Methods 3 and 4 were not significantly different, and Method 4 resulted in a higher average accuracy compared to Method 5. We believe this was because the decision tree method is not the most suited for finding optimal frequency sets based on the number of common sums due to the integer nature of these numbers, which are the number of times the common sums are observed. As a performance measure in an optimization, this metric does not provide very high resolution as it does not entertain fractions and decimals, which are applied to Methods 3 and 4. Method 5, while also utilizing the decision tree mechanism, uses the correlation between input signals as its performance measure in the optimization. This allows a high degree of resolution, as decimals are allowed, providing better outcome of the optimization process. As such, Method 3 and Method 4 do not perform optimally. Table 3 reveals this issue to some extent, where it can be seen that, toward the end, Methods 3 and 4 arrived at the same frequency pair (15.5, 16) after the optimization process even though they were designed to be doing completely opposite tasks.

Method 3 is designed with what we expected to be minimum number of common sums. As it was not optimal, the accuracy was expected to be an underestimate of its potential. However, compared to Method 5, it still yields significantly higher accuracy. Method 4 is designed to maximize the number of common sums. This was expected to yield the lowest accuracy among Methods 3, 4, and 5. As it is not optimal, the resulting accuracy was higher than what was expected.

### 4.2. General SSVEP performance

In terms of time-domain waveform, in Figure 4, we can see that some SSVEP waveforms are highly consistent with the stimulation waveform (e.g., 11 and 12 Hz), whereas some other waveforms are more complex. This is likely because the human brain is a highly nonlinear system that produces complex response to even simple single-frequency stimulation. For the plots that show SSVEPs stimulated by dual-frequency signals, it is expected that there will be more frequency components (interactions between the two input frequencies) in the EEG signal. Thus, when some frequency interactions are dominating the response, the SSVEP waveform may look different to the stimulation waveform.

While the focus of this study was not on boosting the SSVEP performance, we are aware of the difference in performance from our results compared to some studies in the literature for both dual-frequency SSVEP (Liang et al., 2020) and single-frequency SSVEP (Chen et al., 2015). One source of the difference might be attributed to the use of dry electrodes in our study as opposed to wet electrodes commonly used in other studies. It has been shown that there is a 20% accuracy drop when using dry electrodes compared to wet electrodes even though the signals look similar in both time domain and frequency domain (Zhu et al., 2021).

### 4.3. Limitations

Even though both hypotheses were validated, this work was only tested with the selected multi-frequency stimulation method (frequency superposition). This means that the result may vary if a sufficiently different stimulation method which triggers completely different multi-frequency SSVEP patterns were used. We also acknowledge that only the decision tree method was implemented in optimization in this study and it has its own limitations though its effectiveness compared to global search was already proven (Liang et al., 2020). Furthermore, since MFCCA is the only purposefully designed generalized multi-frequency SSVEP decoder, it was used in this study to avoid extensive training and prolonged experiment duration. However, the accuracies from the dual-frequency tests were at a lower level compared to the single-frequency test. This is not considered a problem here because this study focuses on understanding the difference between different frequency set selection methods in multi-frequency SSVEP and the single-frequency test was included for bench-marking purpose only. Nonetheless, this suggests that more effort should be spent on developing generalized multi-frequency SSVEP decoding algorithms to improve the overall performance of multi-frequency SSVEP-based BCIs.

### 4.4. Future work

As part of future work, other optimization methods should be explored to address the potential pitfall in the decision tree when working with the number of common sums. Other optimization strategies regarding the frequency set selection in multi-frequency SSVEP should also be further explored. Last, but not least, a comprehensive comparison between single-frequency and multi-frequency SSVEP should be conducted with finer frequency intervals and larger frequency coverage.

## 5. Conclusion

The results from the frequency set selection study showed that selection by pairs (compared to selection by frequencies) and optimizing (minimizing) the number of common sums in selection by pairs significantly increased the accuracy of the interface. Furthermore, a potential pitfall was observed in the decision tree method in optimizing the number of common sums, which resulted in a sub-optimal result from the optimization process and subsequently a smaller than expected difference between the best and worst case scenarios in the number of common sums optimization.

## Data availability statement

The raw data supporting the conclusions of this article will be made available by the authors, without undue reservation.

## Ethics statement

The studies involving human participants were reviewed and approved by University of Melbourne Human Research Ethics Committee. The patients/participants provided their written informed consent to participate in this study.

## Author contributions

JM, DG, YT, and DO contributed toward the conception and design of the work, data analysis, and interpretation. JM performed data collection and drafted the manuscript. DG, YT, and DO provided critical revisions of the article. All authors reviewed and approved the submitted version.
